# Observation of low-loss broadband supermode propagation in coupled acoustic waveguide complex

**DOI:** 10.1038/srep45603

**Published:** 2017-03-28

**Authors:** Ya-Xi Shen, Yu-Gui Peng, Xin-Cheng Chen, De-Gang Zhao, Xue-Feng Zhu

**Affiliations:** 1 School of Physics, Huazhong University of Science and Technology, Wuhan, Hubei 430074, People’s Republic of China; 2 Innovation Institute, Huazhong University of Science and Technology, Wuhan, Hubei 430074, People’s Republic of China; 3 State Key Laboratory of Functional Materials for Informatics, Shanghai Institute of Microsystem and Information Technology, Chinese Academy of Sciences, 865 Changning Road, Shanghai 200050, People’s Republic of China

## Abstract

We investigate analytically, numerically, and experimentally the low-loss supermode propagation in a coupled acoustic waveguide complex within a broadband. The waveguide complex is implemented with air channels coupled via an ultrathin metafluid layer. We analytically derive the field distribution of incident sound needed for producing acoustic supermodes, and verify the periodically revival propagation in coupled waveguide systems numerically and experimentally. We find out that the supermode wavelength becomes longer for higher mode order or lower frequency. We have also demonstrated the robust propagation of supermodes in broadband. Our scheme can in principle be extended to three dimensions and the ultrasound regime with simplicity and may promote applications of high-fidelity signal transfer in complicated acoustic networks.

Anomalous manipulations of acoustic energy flow have received many attentions during the past decades, such as negative refraction/reflection^1^,^2^,^3^,^4^,^5^, collimation^6^,^7^,^8^, super-resolution imaging^9^,^10^,^11^, cloaking^12^,^13^,^14^,^15^,^16^, rainbow trapping^17^,^18^,^19^, beam acceleration^20^,^21^,^22^, and topological transportation^23^,^24^,^25^,^26^,^27^,^28^, *etc*. The recent advances in acoustical metamaterials and metasurfaces provide a broad platform which enables those intriguing phenomena to be realized in experiments^29^,^30^. Due to diffraction, acoustic devices for anomalous wave-steering in free space are inevitably to be large in size. Therefore, a more promising candidate, *viz*. acoustic waveguide networks, is proposed for device integration, where the size may markedly decrease due to the lack of cut-off in rigid channels^10^,^11^,^25^,^27^. Previous works have shown that sound propagation in waveguide networks can be much more intricate than that in bulk media^25^,^27^. For example, the acoustic waves propagating in one waveguide may easily spread to the whole system through couplings. In practice, such coupling-induced diffraction is undesirable, since the information carried by acoustic signals will be smeared or considerably modified by diffraction during the propagation. It thus should be meaningful, both in physics and in engineering, to explore the possibility to realize non-diffracting acoustic wave propagation for high-fidelity signal transfer in integrated networks.

In this paper, we propose to employ supermodes to implement non-diffracting propagation in one type of acoustic networks, *viz.*, an array of coupled waveguides. The resulting device simply consists of five air channels coupled via four gratings. We rigorously derive the desired field distribution of incident waves for producing acoustic supermodes. Remarkably, the supermode propagations are featured with broadband stability and low loss, since the device has no resonant structures with frequency-dependent responses. We further demonstrate those intriguing properties of acoustic supermodes through proof-of-concept experiments. With the simple design and fabrication, our proposal can be in principle extended to three dimensions and the ultrasound regime, and make a significant step towards the application of high-fidelity transportation of broadband acoustic signals in integrated acoustic networks.

## Results

**Figure 1:**
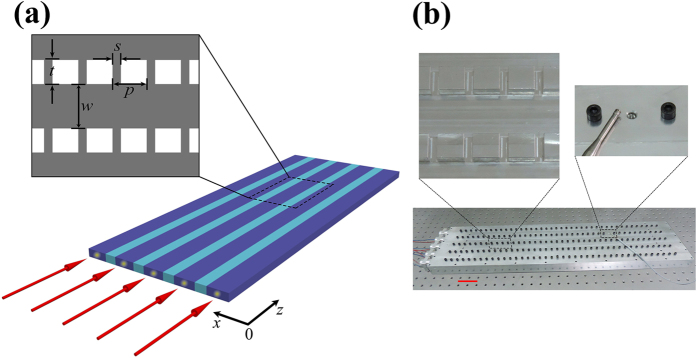
Schematic and Photograph of an acoustic waveguide array. (**a**) Schematic of the studied acoustic waveguide array, where five air channels are segregated by four gratings. The red arrows show the path of incident sound. Inset: Structure details of channels and gratings. The gray and white regions represent air and rigid metal, respectively. (**b**) Photograph of the fabricated acoustic waveguide array. Inset: machined inside channels, inside gratings, and measurement holes. Scale bar, 5 cm.

We start from investigating the acoustic supermode in an array of *N* coupled waveguides based on the coupled-mode theory, with only the nearest-neighbor coupling being considered^31^. The scalar wave equation for sound takes the form of 
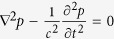
. As shown in Fig. 1(a), we investigate a two-dimensional waveguide complex in *x*-*z* plane. Here, the pressure and speed of sound are expressed into 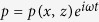 and 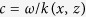, where *k(x, z*) represents the wave number. From the aforementioned equations, we can deduce the governing equation 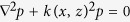. For a linear and scalar system, we separate the variables to have 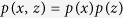 and 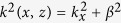, where *β* is the propagation constant along *z* direction. It should be pointed out that *β* > *k(x, y*) for a guiding mode. Thus, *k*_*x*_ should be imaginary, indicating that the field along *x* direction is evanescent. Substituting 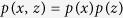 and 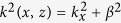 into the governing equation, we will obtain


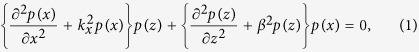


which further leads to 
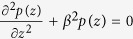
 and 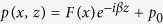. Here, *p*_0_ is a constant that could be chosen to be zero by setting a proper coordinate origin, and *F(x*) is the mode profile along *x* direction. For an array of *N* coupled waveguides, *F(x*) can be expressed into a discrete form of 
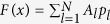
, with *A*_*l*_ being the weight of a propagating mode in one waveguide. Therefore, the mode profile of the whole system can be written into


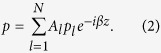


In this work, we only consider the nearest-neighbor coupling (the tight-binding model) and set all waveguides identical and equally spaced. Substituting Eq. (2) into the governing equation, we can finally derive the matrix


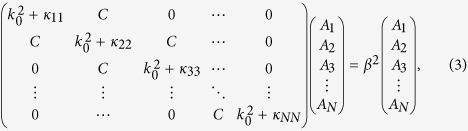


where *k*_0_ is the wave number in free space, *C* is the coupling coefficient between nearest neighbor waveguides, and 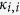 is the self-coupling coefficient for the *i*th waveguide. Due to different boundary conditions, the self-coupling coefficients 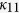, 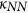 do not equal to 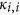

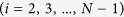
. Therefore, we may define 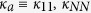, 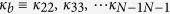, and 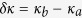. After numerous mathematical deductions, the eigenvalues and eigenvectors of Eq. (3) are derived to be ref. 32


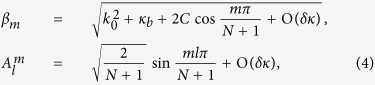


where *m* is the mode order, and *l* is the waveguide number. In Eq. (4), *β*_*m*_ also represents the propagation constant of *m*-ordered supermode. It is interesting that the mode intensity in an air channel will be zero on condition that *ml*/(*N* + 1) is an integer, and the mode intensity will be non-zero for all air channels if *N* + 1 is a prime number. From the above analysis, we derive that acoustic supermodes are actually the eigen-modes of the waveguide complex.

In this paper, the studied waveguide complex comprises five air channels segregated by four gratings (*N* = 5), as illustrated in Fig. 1(a,b). In our design, the channels and gratings are fabricated with Aluminum alloy that is treated as rigid to air. Propagating inside the waveguide complex, acoustic waves in one channel will be allowed to tunnel into the neighboring ones through periodic gratings. Based on the effective medium approach, acoustic gratings can be equivalent to metafluid layers with non-dispersive bulk modulus and density tensor in the long wave condition^10^,^11^. In respect that there are no local resonances as well as weak damping in air channels and metafluid layers, the wave propagation in this coupled waveguide array is featured with low energy loss in broadband. For the structural parameters, the thickness, slit width, and slit period of gratings are *t* = 1.4 cm, *s* = 0.5 cm, and *p* = 2 cm, respectively. The width of air channels *w* = 2.6 cm. According to Eq. (4), we can predict that there are five supermodes supported by the waveguide array since *N* = 5, and analytically obtain the weight factors of all air channels for each supermode by ignoring *δκ*.

**Figure 2:**
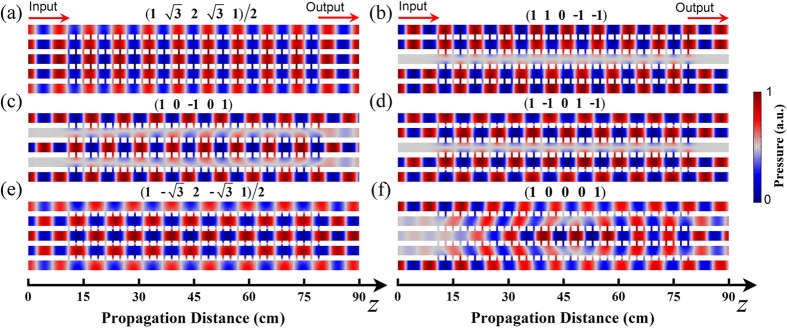
Pressure fields of different propagating modes. (**a**–**e**) Normalized pressure field distributions of five different supermodes at *m* = 1, 2, 3, 4, 5, respectively. The weight factors of all air channels for each supermode are appended on top of the corresponding panels. (**f**) Normalized pressure field distribution when the input does not form a supermode. The operation frequency *f* = 4 kHz.

Next, we simulate the sound propagation in an array of five coupled waveguides in Fig. 2(a–f), where the thermo-viscous damping effect is taken into consideration (see the Method section). In the numerical simulation, the total waveguide length is 90 cm. There are 35 slits connecting two adjacent channels. For convenience, we number the channels as 1, 2, 3, 4, 5 from below. Figure 2(a–e) show the normalized pressure field distributions for five different supermodes at *m* = 1, 2, 3, 4, 5, where the operation frequency *f* = 4 kHz and the excitation condition is determined by Eq. (4). The results clearly present the stable propagation of acoustic supermodes, where the field pattern is periodically repeated along *z* axis. We find out that the supermodes can be classified into symmetric and anti-symmetric ones for odd and even orders, respectively. To be specific, in Fig. 2(a,b), the supermode at *m* = 1 takes the form of 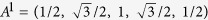, while the one at *m* = 2 is 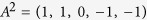. From the field maps, we note that the waveguides at (*m, l, N*) = (2, 3, 5), (3, 2, 5), (3, 4, 5), and (4, 3, 5) are nearly “dark” due to destructive interferences, in well agreement with the aforementioned criterion that *ml*/(*N* + 1) should be an integer for 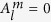. It should be pointed out that the acoustic supermode can be easily excited in experiments, since there are only two types of coupling between adjacent air channels, *viz*., in-phase and out-of-phase couplings. Hence, we only need a simple voltage inverter circuit with tunable current amplitude and 0(or *π*)-phase to drive a set of emitters. By contrast, an arbitrarily chosen input (1, 0, 0, 0, 1) is sent into the coupled waveguide array. As shown in Fig. 2(f), after propagating through a certain distance, acoustic waves are clearly spreading to all channels via the coupling-induced diffraction, with pressure field pattern considerably modified at the output. In this case, the information carried by acoustic waves at the input can hardly be extracted at the output.

**Figure 3:**
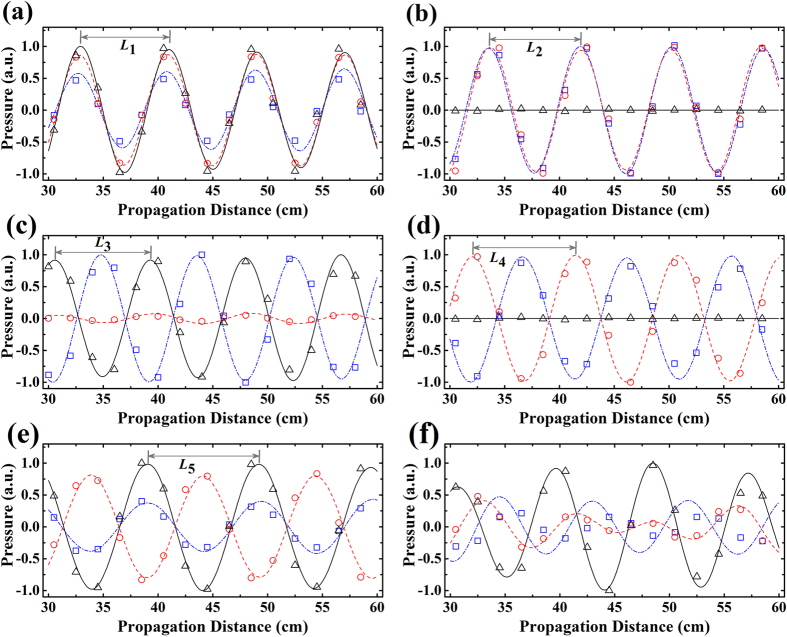
Numerical simulation and experimental measurement. (**a**–**f**) Simulated and measured pressure field distributions along the central line of channels 1, 2, and 3 from *z* = 30 cm to *z* = 60 cm, corresponding to the cases of Fig. 2(a–f), respectively. The blue dot dash line, red dashed line, and black solid line are the simulation data of channels 1, 2, and 3, while the blue squares, red circles, and black triangles are the measured data of channels 1, 2, and 3 in experiments. In (**a**–**e**), the wavelengths of five supermodes at *m* = 1, 2, 3, 4, 5, are marked by *L*_1_, *L*_2_, *L*_3_, *L*_4_, and *L*_5_, respectively.

For characterizing wave propagations in the coupled waveguide array in details, we extract the waveforms of channels 1, 2, and 3 from the data in Fig. 2(a–f), as plotted by the curves in Fig. 3(a–f). Figure 3(a–e) show the waveforms of different supermodes from *z* = 30 cm to *z* = 60 cm at *m* = 1, 2, 3, 4, 5, respectively. For all the supermodes, sound in each air channel bears a stable propagation with the amplitude unchanged. As an example, for the supermode at *m* = 1 in Fig. 3(a), we clearly see the perfect sinusoidal waveforms in channels 1 (blue dot dash line), 2 (red dashed line), and 3 (black solid line), with the amplitude ratio being 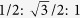 and phase locked. In the aforementioned theoretical part, we have shown that the supermode in a coupled waveguide array takes a modulated plane wave form of 
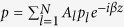
. Therefore, it is predicted that the modal field will revive at intervals of *L*_*m*_ = 2*π*/*β*_*m*_, *viz*., supermode wavelength. From Eq. (4), *β*_*m*_ becomes smaller as *m* increases. We will obtain that the higher-ordered supermode has a longer wavelength. In Figure 3(a–e), the supermode wavelengths are numerically calculated to be *L*_1_ ≈ 8.10 cm, *L*_2_ ≈ 8.36 cm, *L*_3_ ≈ 8.78 cm, *L*_4_ ≈ 9.54 cm, and *L*_5_ ≈ 10.33 cm, which agrees well with the theoretical prediction. It should also be mentioned that we can further derive the coupling coefficient between nearest neighbor waveguides *C* and the self-coupling coefficient *κ*_*b*_ from the calculated supermode wavelengths *L*_*m*_. In our case, we have *C* ≈ 7.67 × 10^−2^ cm^−2^ and 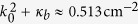. Figure 3(f) displays the waveform of a normal propagation mode in the coupled channels, featured with constantly changing wave amplitude and phase differences between two arbitrary channels.

To demonstrate the existence of acoustic supermodes experimentally, we have fabricated the sample of waveguide complex with five coupled air channels, and measured the pressure field through perforated holes, as shown in Fig. 1(b). For each air channel, there are 40 holes equally spaced by 2 cm. The details of experimental measurements are presented in the Method section. The measured results are shown by the dots in Fig. 3(a–f), where the blue squares, red circles, and black triangles are the measured data of channels 1, 2, and 3 after normalization. We clearly see that the experimental results are in very good agreement with numerical simulations.

**Figure 4:**
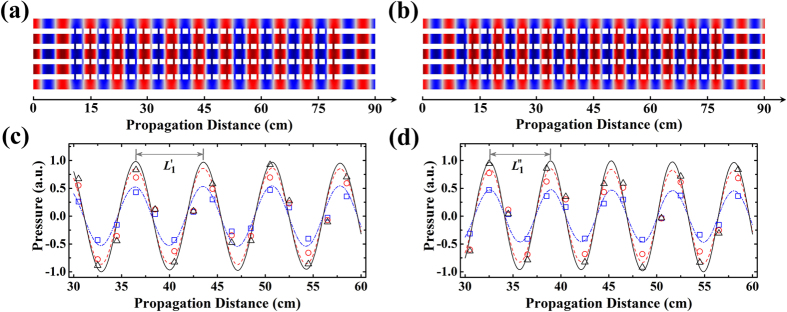
Robustness of acoustic supermodes at different frequencies. (**a**,**b**) Normalized pressure field distributions of the 1st-ordered supermodes at 4.5 kHz and 5 kHz, respectively. As predicted by Eq. (4), both supermodes take the same form of 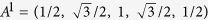. (**c**,**d**) Simulated and measured pressure field distributions along the central line of channels 1, 2, and 3 from *z* = 30 cm to *z* = 60 cm, corresponding to the cases of (**a**) and (**b**), respectively. The blue dot dash line, red dashed line, and black solid line are the simulation data of channels 1, 2, and 3, while the blue squares, red circles, and black triangles are the measured data of channels 1, 2, and 3 in experiments.

We would also like to point out that the formation of acoustic supermode has nothing to do with frequency, as theoretically unveiled by Eq. (4). To verify this, we simulate the propagation of the 1st-ordered supermodes at two different frequencies of 4.5 kHz and 5 kHz, respectively, as shown in Fig. 4(a,b). The maps of pressure field distribution display the stable propagation of supermodes, in respect that the output signal is almost the same as the input. From the simulation, we can obtain the supermode wavelengths of the 1st-ordered supermodes at 4.5 kHz and 5 kHz, *viz*., 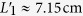 and 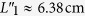, which shows that the supermode wavelength becomes longer at lower frequencies. In Figure 4(c,d), the experimental results agree with the simulation quite well, with both indicating that the designed waveguide complex can effectively support the acoustic supermode propagation in broadband.

**Figure 5:**
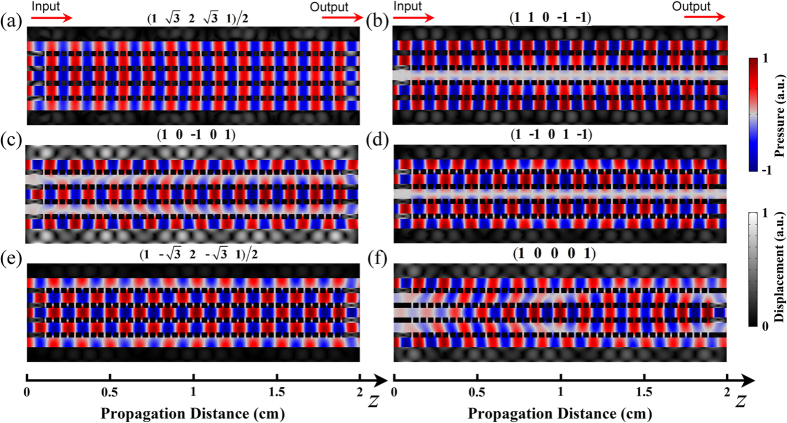
Pressure fields of different propagating modes in the ultrasound regime. (**a**–**e**) Normalized pressure field distributions of five different supermodes at *m* = 1, 2, 3, 4, 5, respectively. The weight factors of all water channels for each supermode are appended on top of the corresponding panels. (**f**) Normalized pressure field distribution when the input does not form a supermode. The operation frequency *f* = 1.02 MHz.

At last, we show that our technique can be applied in the ultrasound regime, which might be useful in high-fidelity broadband underwater communication. In the proof-of-concept simulation, we employ Steel (mass density *ρ* = 7.80 × 10^3^ Kg/m^3^; velocity of longitudinal waves *c*_*l*_ = 6100 m/s; velocity of transverse waves *c*_*t*_ = 3300 m/s) instead of Aluminum alloy to reduce solid-liquid coupling. The operation frequency is chosen to be 1.02 MHz. For the structural parameters scaled by approximately 0.022, the thickness, slit width, and slit period of gratings are *t* = 0.308 mm, *s* = 0.11 mm, and *p* = 0.44 mm, respectively. The width of air channels *w* = 0.572 mm. We simulate the ultrasound propagation in the coupled waveguide complex in Fig. 5(a–f), where the thermo-viscous damping effect and solid-liquid coupling are taken into consideration. Even though there exist some field disturbances due to solid-liquid interaction, our results still reflect the characteristic propagation of acoustic supermodes, where the field pattern is periodically repeated along *z* axis.

## Discussions

In summary, we have investigated the supermode propagation in coupled acoustic waveguides analytically, numerically, and experimentally. The experimental results agree well with the theoretical prediction and numerical simulation. The proposed device bears the advantages of easy fabrication, small fingerprint, low-loss and broadband performance, as well as the potential to further extend into higher dimensions and the ultrasound regime, which is very promising in various practical applications, such as underwater acoustic communication, ultrasound imaging, and sensing, *etc*. Our study may pave the way to explore other types of anomalous wave manipulation in integrated acoustic networks.

## Methods

### Numerical simulation

Throughout this paper, we consider the thermo-viscous damping in all numerical simulations by employing thermo-acoustics module of COMSOL Multiphysics^TM^ 5.2. A perfectly matched layer is added at the right side to prevent reflections. In the ultrasound simulation, the solid-fluid interaction is also take into consideration.

### Experimental measurement

In the experiment, we employ a multi-function waveform generator (RIGOL DG1032Z) and a stable power amplifier (AOSIBAO A8 HIFI) to output multiple locked sinusoidal electrical signals, and then use a connected full-range driver (HiVi B1S) to convert the electrical signals into sound. The pressure field is measured by a 1/8-inch diameter Brüel&Kjær Type-LAN-XI-3160 condenser microphone. All data are recorded and processed with Brüel&Kjær PULSE 3160-A-042 4-channel analyser. During the field measurement, other unmeasured holes are plugged with screws to prevent sound leakage.

## Additional Information

**How to cite this article**: Shen, Y.-X. *et al*. Observation of low-loss broadband supermode propagation in coupled acoustic waveguide complex. *Sci. Rep.* **7**, 45603; doi: 10.1038/srep45603 (2017).

**Publisher's note:** Springer Nature remains neutral with regard to jurisdictional claims in published maps and institutional affiliations.
